# Phytochemical Analysis, In Vitro and In Vivo Evaluation of *Ficus altissima* Extract‐Based Ointment and Hydrogel on Wound Healing

**DOI:** 10.1155/bmri/5137800

**Published:** 2026-04-28

**Authors:** Hossein M. Elbadawy, Aryam S. Alharbi, Ahmed K. B. Aljohani, Asalah B. Alhazmi, Manhal N. Hudhayri, Salma A. Ibrahim, Israa B. Almuwalld, Maya A. Alhazmi, Shuruq M. Almohammadi, Ahmed Aldhafiri, Heba M. Eltahir, Mekky M. Abouzied, Hamad Alrbyawi, Mohamed R. Kamel, Fahd M. Abdelkarem

**Affiliations:** ^1^ Department of Pharmacology and Toxicology, College of Pharmacy, Taibah University, Madinah, Saudi Arabia, taibahu.edu.sa; ^2^ Health and Life Research Center, Taibah University, Madinah, Saudi Arabia, taibahu.edu.sa; ^3^ College of Pharmacy, Taibah University, Medina, Saudi Arabia, taibahu.edu.sa; ^4^ Pharmacognosy and Pharmaceutical Chemistry Department, College of Pharmacy, Taibah University, Madinah, Saudi Arabia, taibahu.edu.sa; ^5^ Department of Pharmacognosy, Faculty of Pharmacy, Galala University, Suez, Egypt, gu.edu.eg; ^6^ Department of Pharmaceutics and Pharmaceutical Industries, College of Pharmacy, Taibah University, Madinah, Saudi Arabia, taibahu.edu.sa; ^7^ Department of Pharmacognosy, Faculty of Pharmacy, Al-Azhar University, Assiut, Egypt, azhar.edu.eg

**Keywords:** anti-inflammatory, *Ficus altissima*, ointment-based formulation, phytochemical characterization, wound healing

## Abstract

**Objective:**

Wound healing is a sequential mechanism that occurs in four successive stages: hemostasis, inflammation, proliferation, and remodeling. Delayed wound healing and the subsequent complications can be observed in poorly controlled chronic diseases and immunocompromised patients. This study focused on the wound‐healing activity of *Ficus altissima* extract‐based formulations, exploring their phytochemical and biological properties.

**Methods:**

Phytochemical characterization was conducted using HPLC, GC–MS, and spectroscopic methods for quantification of phenolic compounds and flavonoids. Disk diffusion was utilized to test the antimicrobial effects of the extract, and antioxidant activity was measured using DPPH assay. Anti‐inflammatory activity was assessed by the quantification of NF‐*κ*B and TNF‐*α* levels in LPS‐stimulated RAW 264.7 cells. An ointment and hydrogel were prepared from *F. altissima* aerial parts and tested for their wound‐healing effect using skin wound‐healing model in rats.

**Results:**

Phenolic acids, flavonoid compounds, fatty acids, and sugar derivatives were detected in *F. altissima* extract with total phenolic and total flavonoid contents equal to 144.81 mg·GAE g^−1^ and 29.36 mg·QE g^−1^, respectively. *F. altissima* downregulated TNF‐*α* and NF‐*κ*B expression and inhibited the microbial growth of various microorganisms. Both formulations improved wound closure, with the ointment showing superior results within 10 days.

**Conclusions:**

*F. altissima* extract, particularly in an ointment form, effectively promoted wound healing and tissue regeneration, and this effect can be linked to the detected phenolic acids and flavonoids with anti‐inflammatory, antioxidant, and antimicrobial properties.

## 1. Introduction

Open wounds induce a series of events that include inflammation, oxidative stress, and microbial contamination that can potentially affect wound closure and restoration of normal skin integrity [1, 2]. Wounds that result from abrasions, cuts, or punctures differ in severity and trigger a four‐stage healing process: hemostasis, inflammation, proliferation, and remodeling [3]. Hemostasis is the first immediate response to vascular injury, which results in blood vessel constriction and formation of a blood clot to stop bleeding. Next is the inflammatory response, where immune cells come into action to eradicate pathogens, thereby preventing and controlling the spread of infection. After that, the proliferation phase begins with angiogenesis and fibroblast activation to produce collagen for tissue repair. Finally, remodeling strengthens and restores the new tissue′s structure [4]. Poor management of open wounds can lead to delayed healing, chronic infections, tissue necrosis, or sepsis, often worsened by infections and host immune or inflammatory responses [5, 6].

Natural extracts and their active constituents are traditionally used for maintaining health, boosting immunity, preventing infections, and promoting healing [7]. Plant‐derived natural products provide antioxidants, antimicrobial agents, and anti‐inflammatory substances that aid in tissue regeneration [8]. They offer diverse active compounds, including flavonoids, carotenoids, terpenoids, phenolics, tannins, alkaloids, steroids, and saponins, which support healing through various mechanisms [9]. It is worth noting that plants are widely used for their affordability, availability, and minimal side effects [10]. Over 400 plant species show potential for wound healing; the WHO reported that 80% of the global population uses natural products for treating many disorders and for primary healthcare [11, 12]. Integrating these remedies into modern medicine offers promising solutions for wound management and improved health outcomes [9]. Wound‐healing agents are a growing area of research in modern medicine, yet more effective treatments remain a challenge for researchers [13].

The *Ficus* genus is classified as a member of the Moraceae family, comprising over 800 species mainly found in tropical regions and valued for their economic and nutritional benefits [14, 15]. Many *Ficus* species are traditionally used to treat wounds, burns, and bruises [16]. They contain diverse bioactive compounds such as tannins, phenolic compounds, saponins, carbohydrates, alkaloids, proteins, ketones, flavonols, coumarins, pentacyclic triterpenes, triterpenoids, esters, polysaccharides, lignans, terpenoids, sterols, flavonoids, glycosides, and vitamins C, E, and K [17]. These compounds exhibit antibacterial, hepatoprotective, antidiabetic, anti‐inflammatory, nephroprotective, antityrosinase, cardioprotective, antitumor effects, along with other pharmacological properties [18]. *Ficus altissima* (*F. altissima*) is a flowering plant that is used in Chinese medicine for treatment of cardiovascular diseases and dementia [19]. The roots of the plant are used to aid blood circulation and detoxification [20], whereas the tannins from its leaves provide antioxidant and antityrosinase effects [21]. Its fruits also show anti‐inflammatory and anticancer activity, whereas its bark and leaves have been investigated for treating skin disorders [19–22].

Although no previous studies have explored the wound‐healing potential of *F. altissima* and only few reports have considered its phytochemical composition, its content of bioactive compounds prompted our interest. In contrast to previous studies on other *Ficus* species, the present work offers a comprehensive and integrated investigation of *F. altissima* methanolic leaf extract. This study systematically characterizes its phytochemical profile using HPLC and GC–MS analyses, evaluates its wound‐healing potential through combined in vitro assays, in vivo excision wound model, and topical formulations (ointment and hydrogel), and further explores the underlying molecular mechanisms by examining the expression of key inflammatory mediators through gene expression analysis. This integrated experimental design clearly highlights the novelty and scientific significance of the present study.

## 2. Materials and Methods

**SCHEME 1:**
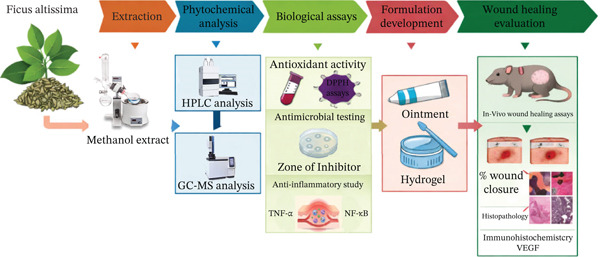
Flowchart illustrating the overall experimental workflow for evaluation of wound‐healing potential of *F. altissima* and their phytochemical characterization.

The overall experimental workflow and study design applied in the phytochemical investigation and evaluation of wound‐healing potential of *F. altissima* extract are summarized in Scheme 1.

### 2.1. Plant Material and Preparation of *F. altissima* Extract

The aerial parts of *F. altissima* were obtained from the “Medicinal, Aromatic and Poisonous Plants Research Center” in Riyadh in September 2024, Kingdom of Saudi Arabia (KSA). The plant′s identity was confirmed by experts at the Faculty of Pharmacy, Taibah University. A total of 500 g of dried aerial parts was finely chopped and subjected to extraction using 85% methanol (1 L, repeated three times) under ambient conditions [23, 24]. The extract was then filtered and concentrated as the solvent was evaporated utilizing a rotary evaporator set at 35°C under reduced pressure. This process resulted in 52.0 g of a dried crude extract with a brownish appearance.

### 2.2. Phytochemical Investigation

#### 2.2.1. HPLC Qualitative Estimation of Flavonoids and Phenolic Compounds

Ten phenolic compounds and 10 flavonoids were selected as reference compounds, with purity certified at 99%. The analysis was performed using an Agilent 1260 series system (United States) with a Zorbax Eclipse plus C8 column (4.6 × 250 mm, 5 *μ*m). The UV detector was adjusted to 280 nm. The mobile phase consisted of water (A) and 0.05% TFA in acetonitrile (B) at a flow rate of 0.9 mL/min. The gradient program was as follows: 0 min (82% A); 0–1 min (82% A); 1–11 min (75% A); 11–18 min (60% A); 18–22 min (82% A); and 22–24 min (82% A). The multiwavelength detector was set to 280 nm. A 5‐*μ*L injection volume was used for each sample, and the column temperature was kept at 40°C throughout the analysis [25, 26].

#### 2.2.2. GC–MS Analysis of the Crude Extract

*F. altissima* crude extract was treated with sodium hydroxide (NaOH), followed by acidification with hydrochloric acid (HCl) and subsequent extraction using ethyl acetate. The solvent was then evaporated under a nitrogen stream at 40°C. The resulting sample was dissolved in 50 *μ*L of a mixture of [bis (trimethylsilyl) trifluoroacetamide (BSTFA) and trimethylchlorosilane (TMCS, 99:1)], then combined with 50 *μ*L of pyridine to accelerate the derivatization of functional groups into trimethylsilyl (TMS) derivatives for gas chromatography (GC) analysis. The GC–MS system (Agilent, United States) was equipped with a DB‐5MS column (30 × 0.25 mm internal diameter, 0.25‐*μ*m film thickness). Hydrogen gas was employed as the carrier. The analysis was performed under the gradient temperature program with a 1‐*μ*L injection volume. The electron impact ionization was set at 70 eV, covering a mass‐to‐charge (m/z) range of 50–800, using a 7.3‐min solvent delay. To identify individual components, retention times and spectral fragmentation patterns were compared with reference values [25, 26]. Wiley and NIST Mass Spectral Library databases were used for reference values.

#### 2.2.3. Determination of the Total Phenolic Content (TPC)

The total content of phenolic compounds in *F. altissima* extract was assessed with the Folin–Ciocalteu colorimetric method [25–27]. In summary, 1 mg of the crude extract was dissolved in 2 mL of methanol, and 500 *μ*L of this solution was combined with 2.5 mL of diluted Folin–Ciocalteu reagent (Merck, Germany) and 2.5 mL of sodium carbonate (75 g/L). The mixture underwent vigorous agitation for 10 s and was then left to stand for 2 h at 25°C. Following incubation, the absorbance was measured at 765 nm using a Milton Roy Spectronic 1201 spectrophotometer (Milton Roy, United States). The phenolic contents were calculated as gallic acid equivalents (GAE) (mg/g dry extract).

#### 2.2.4. Determination of Total Flavonoid Content (TFC)

Quantification of flavonoid pool in *F. altissima* extract was determined following the modified AlCl₃ colorimetric method as previously reported [28]. One milligram portion of the crude extract was dissolved in 2 mL of methanol within a 10‐mL volumetric flask. Solutions of 5% NaNO_3_, 5% NaOH, and 7% AlCl_3_ were prepared using water in 25‐mL volumetric flasks. A 200‐*μ*L aliquot of the extract was transferred into a sealed glass vial, and 75 *μ*L of 5% NaNO_3_ was added. After incubation for 5 min at room temperature, 1.25 mL of AlCl_3_ solution and 0.5 mL of NaOH solution were added to the vial, and the mixture was sonicated and incubated for 5 min at room temperature. After the incubation, the absorbance was measured at 510 nm against a methanol blank using quercetin as a standard. The flavonoid content of the extract was quantified based on a quercetin calibration curve and expressed as milligrams of quercetin equivalents (QE) per gram of dry extract (mg/g dry extract).

#### 2.2.5. Determination of Free‐Radical Scavenging Activity

The antioxidant potential of *F. altissima* crude extract was assessed using the DPPH (2,2‐diphenyl‐1‐picrylhydrazyl‐hydrate) assay to evaluate its free‐radical scavenging activity, with vitamin C serving for positive control [29]. Briefly, a range of concentrations of the *F. altissima* extract were obtained through serial dilution. Reactions were initiated through adding 200 *μ*L of either the crude extract solution or ascorbic acid (positive control) to 2000 *μ*L of a 0.1 mM DPPH solution. Reaction contents were thoroughly mixed and incubated in the dark at room temperature for 30 min. Following incubation, absorbance was recorded at 570 nm using a spectrophotometer. The percentage of free‐radical scavenging activity was determined using the following formula:

DPPH radical scavenging activity=Abs control−Abs sample/Abs control ×100.

All experiments were performed in three replicates (*n* = 3), and the average from the three repetitions was used to express the results in the form of mean ± SEM IC_50_ values. The IC_50_ was determined from the concentration–response curve with GraphPad PRISM.

### 2.3. Antimicrobial Activity Assay

#### 2.3.1. Antimicrobial Activity via Disk Diffusion Assay

The antimicrobial activity of *F. altissima* crude extract was evaluated using the agar well diffusion method [30]. One hundred microliter aliquots of the following microbial suspensions—*Bacillus subtilis* (ATCC 6633), *Staphylococcus aureus* (ATCC 6538), *Escherichia coli* (ATCC 8739), *Klebsiella pneumoniae* (ATCC 13883), *Candida albicans* (ATCC 10221), and *Aspergillus niger* (ATCC 16888)—were uniformly spread on Mueller‐Hinton agar for bacterial strains or Sabouraud dextrose agar for fungal strains. Wells measuring 6 mm in diameter were made in the agar, and 50 *μ*L of *F. altissima* crude extract (50 mg/mL) was introduced into each well. Gentamicin (10 *μ*g/mL) was used as a positive control for bacterial strains, whereas fluconazole (10 *μ*g/mL) was used as a positive control for fungal strains. Incubation was carried out at 37°C for 24 h for bacterial cultures and at 25°C for 48 h for fungal cultures. The antimicrobial activity was assessed by measuring the diameter of the inhibition zones (mm), with classification into four categories: very strong (> 20 mm), strong (10–20 mm), medium (5–10 mm), and no response (<5  mm) [31].

#### 2.3.2. Determining Minimal Inhibitory Concentration (MIC)

The MIC of the crude extract of *F. altissima* was determined using the broth microdilution method according to the guidelines of the Clinical and Laboratory Standards Institute (CLSI). To conduct the assay, a pure culture of the target microorganism is first incubated overnight. This culture is subsequently diluted in nutrient broth, specifically Tryptic Soy Broth (TSB), to obtain a standardized inoculum with a final density ranging from 1 × 10^5^ to 1 × 10^6^ colony‐forming units per milliliter (CFU/mL). A primary stock solution of the crude *F. altissima* extract is formulated at a concentration roughly 100 times higher than the anticipated MIC. From this stock, a series of twofold dilutions (spanning 1000 *μ*g/mL down to 1.95 *μ*g/mL) is prepared directly in TSB within the wells of a 96‐well microtiter plate. For quality control, negative control is incorporated. This consists of the identical twofold dilution series (1000–1.95 *μ*g/mL) but without the addition of the test organism, serving to establish a baseline and correct for any inherent turbidity caused by the extract itself. Following setup, the microtiter plates are placed in incubation under specified conditions, generally at 35 ± 2°C for a period of 16 to 20 h. After incubation, the presence or absence of growth is evaluated by observing turbidity. This assessment can be performed by unaided eye and by measuring optical density (OD) at a wavelength of 630 nm using an instrument such as the BioTek 800 TS microplate reader. Such devices are acceptable for aiding in the interpretation of microdilution tests and documenting outcomes, provided that they do not hinder the ability to distinguish growth in any well. The MIC endpoint is established by comparing the level of growth observed in wells containing the *F. altissima* extract against the growth seen in the negative control wells included within the same test batch.

### 2.4. In Vitro Anti‐Inflammatory Activity

#### 2.4.1. Reagents and Chemicals

Lipopolysaccharide (LPS) was obtained from Sigma‐Aldrich (St. Louis, Missouri, United States). Fetal bovine serum (FBS) and Dulbecco′s Modified Eagle′s Medium (DMEM) were sourced from Hyclone, a division of General Electric Healthcare Life Sciences (Mississauga, Canada). The penicillin–streptomycin (P/S) solution was procured from Solarbio Life Sciences (Beijing, P.R. China).

#### 2.4.2. In Vitro Cytotoxicity Study

For the evaluation of cell viability, in vitro cell culture assay was used. RAW 264.7 murine macrophages were seeded in cell culture grade 96‐well plates at a density of 4 × 10^3^ cells/well and treated with different concentrations of *F. altissima* extract for 24 h. Following this, a 5 mg/mL solution of 3‐(4,5‐dimethylthiazol‐2‐yl)‐2,5‐diphenyltetrazolium bromide (MTT; Sigma‐Aldrich) was introduced, and the cells were incubated at 37°C with 5% CO₂. After 4 h, the medium was discarded, and DMSO was added to solubilize the formazan product. The absorbance of the resulting solution was then recorded at 490 nm utilizing a UVmax kinetic plate reader. All concentrations were measured in triplicate.

#### 2.4.3. Cell Culture

RAW 264.7 cells were maintained in DMEM supplemented with 10% FBS and 1% P/S at 37°C with 5% CO₂ in 24‐well plates (3 × 10^5^ cells/well). The cells were then assigned to different experimental conditions: untreated control with supplemented medium, medium supplemented with LPS (1 *μ*g/mL) for 24 h to induce inflammation, or medium containing both LPS (1 *μ*g/mL) and *F. altissima* extract (100 *μ*g/mL). To assess anti‐inflammatory properties, cells were pretreated with *F. altissima* for 12 h, then exposed to LPS (1 *μ*g/mL) for an additional 24 h to induce an inflammatory response.

#### 2.4.4. RT‐qPCR Analysis

To investigate the anti‐inflammatory effects of *F. altissima*, RAW 264.7 cells treated with LPS (1 *μ*g/mL/mL) or LPS plus extract (100 *μ*g/mL) for 24 h were rinsed once with sterile PBS then incubated with lysis buffer for 5 min (200 *μ*L/well of Bio‐Rad iScript Sample Preparation Reagent, Bio‐Rad SPR; 170‐8898). According to the manufacturer′s protocol, cell lysates were collected after the incubation without interrupting the cell remnants and were then used for RNA extraction and gene expression analysis of tumor necrosis factor‐alpha (TNF‐*α*) and nuclear factor‐kappa B (NF‐*κ*B) by using RT‐qPCR (Table S1). All samples were measured in triplicates at the (100 *μ*g/mL) concentration.

Relative gene expression was evaluated with the following fold change equation:

Fold change=2−ΔΔCT

where ΔΔCT = [(CT of the gene of interest − CT of the internal control) for sample A] − [(CT of the gene of interest − CT of the internal control) for sample B]; sample A refers to treated cells (RAW 264.7 cells + LPS), and sample B refers to untreated cells (RAW 264.7 cells) [32].

### 2.5. Preparation of the Topical Formulations

#### 2.5.1. Hydrogel Formulation of Crude Extract (*F. altissima* hydrogel [FAHG])

A hydrogel formulation containing 1% w/w of *F. altissima* crude extract was prepared. Hydrogel base was prepared by dissolving 1 g of carbopol 934 in 50‐mL distilled water at 40–45°C followed by the addition of 0.2 g of methylparaben with continuous stirring, then kept overnight. After that, 50 mL of distilled water was stirred into the mixture followed by addition of three to five drops of triethanolamine until the hydrogel formation. For the hydrogel formulation of *F. altissima* extract (1% w/w), 0.5 g of the extract was mixed with 49.5 g of the hydrogel base prepared.

#### 2.5.2. Ointment Formulation of Crude Extract (FAO)

To formulate 1% w/w FAO, 0.5 g of *F. altissima* extract was blended with 49.5 g of an ointment base consisting of beeswax, white soft paraffin, and liquid paraffin. The preparation followed the fusion method, where the extract was introduced into the melted base at 45°C in a water bath and stirred continuously until a uniform dispersion was achieved.

#### 2.5.3. Physical Examination

Visual examination of the hydrogel and ointment formulations was conducted to assess homogeneity, color, and consistency.

#### 2.5.4. pH Measurement

The pH of the hydrogel was measured using a digital pH meter (pH Tutor, Eutech Instruments) by fully immersing glass electrodes into the gel system to ensure complete contact [33]. For the ointment formulation, approximately 1 g of sample was dispersed in 50 mL of water and heated in a water bath at 60°C–70°C. The pH was then determined using a pH meter (pH Tutor, Eutech Instruments). Measurements were performed in triplicate, and the average of three readings was recorded.

#### 2.5.5. Viscosity

The viscosity of the formulations was evaluated using a Brookfield viscometer (S‐62 spindle, model LVDV‐E) at 25°C, operating at a spindle speed of 12 rpm.

#### 2.5.6. Spreadability

The spreadability of FAHG and FAO was assessed by placing 1 g of the formulation onto a glass slide and covering it with another identical slide. A specific weight was applied to the slides, and the time (in seconds) required for the upper slide to completely detach from the lower one was recorded. A shorter duration indicated improved spreadability [34, 35]. The spreadability (g·cm/s) was determined using the following formula:

S=M×L/T

where: *S* = spreadability, *M* = weight tied to upper slide, *L* = length of glass slide, and *T* = time taken for the upper slide to completely separate from the lower slide.

### 2.6. In Vivo Wound‐Healing Assay

#### 2.6.1. Animals and Grouping

Thirty‐six adult Sprague–Dawley rats, aged 8 weeks and weighing 200–250 g, were housed at the animal house facility at the College of Pharmacy, Taibah University, KSA. Animal study and in vivo experimental procedures were reviewed and approved by “*the Research Ethics Committee*” at the College of Pharmacy, Taibah University (COPTU‐REC‐127‐20251002) and performed according to the ARRIVE (Animal Research: Reporting of In Vivo Experiments) guidelines [36]. For acclimatization, all animals were housed individually for 1 week under controlled laboratory conditions, maintaining a temperature of 22°C ± 2°C, humidity levels of 50%–60%, and a 12:12‐h light–dark cycle in an air‐conditioned facility. Rats (all males) were provided with standard rodent chow and water ad libitum, with wood shavings for bedding. Following acclimatization, they were randomly allocated into four experimental groups to assess the impact of different treatments on wound healing postinduction. Group 1 (Negative control group): Animals received no treatment, and wounds were covered only with sterile gauze. Group 2 (Reference ointment group): Animals were given a once‐daily application of the commercially available wound‐healing ointment (Mebo) containing *β*‐sitosterol ([0.25%] in a base of sesame oil and beeswax). Group 3 (Ointment‐treated group): Animals received a single daily topical application of FAO. Group 4 (Hydrogel‐treated group): Animals received a single daily topical application of FAHG. Twenty‐four hours after the first treatment, three rats per group were euthanized for histopathological and immunohistochemical analysis, whereas the remaining six rats in each group completed the study.

#### 2.6.2. Wound‐Healing Model

Prior to the induction of wounds, the rats were anesthetized via intraperitoneal injection of a ketamine (80 mg/kg) and xylazine (10 mg/kg). The dorsal area of each rat was shaved and sanitized with 70% ethanol, after which two full‐thickness circular wounds (10 mm in diameter) were created on either side of the dorsal midline using a heated biopsy punch. The assigned treatments were applied topically once daily, and the wounds were covered with sterile gauze to maintain site protection and ensure uniform exposure. Wound progression was monitored and photographed on days 0, 3, 6, 10, and 15 postwounding, with measurements and analysis performed using ImageJ software. The percentage of wound contraction was calculated using the following formula [37]:

%Wound contraction=Wound area on Day 0−Wound area on Day n/Wound area on Day 0×100.

#### 2.6.3. Histopathological Assessment of Wound Area

Twenty‐four hours after the first treatment, three rats from each group were euthanized, and by the end of the experimental period (Day 21), all remaining rats were humanely euthanized using CO₂ inhalation while still under anesthesia. Skin specimens including the wound area (wounded skin from Day 2 and healed skin tissues from Day 21) were carefully excised, fixed in a 10% formalin solution, serially dehydrated, and embedded in paraffin. Thin sections, each measuring 5 *μ*m in thickness, were then rehydrated and stained with hematoxylin and eosin (H&E) for histological assessment of tissue integrity.

#### 2.6.4. Immunohistochemistry Staining for Vascular Endothelial Growth Factor (VEGF)

Paraffin sections from the wound area were deparaffinized and rehydrated. Then, they were immunostained for VEGF using a monoclonal antibody against VEGF (Santa Cruz Biotech. Inc., Santa Cruz, United States) according to the previously published method [38]. Sections were photographed using a light microscope and blindly evaluated by a specialized histopathologist.

### 2.7. Statistical Analysis

ImageJ software was used for wound area measurements, whereas statistical analysis was conducted using GraphPad Prism (Version 8). The results were expressed as mean ± standard deviation (SD). Group differences were evaluated using a one‐way analysis of variance (ANOVA), followed by Tukey′s post hoc multiple comparison test., with statistical significance set at *p* < 0.05.

## 3. Results

### 3.1. HPLC Qualitative Estimation of Flavonoids and Phenolic Compounds

HPLC analysis of *F. altissima* crude extract indicated the existence of the following nine phenolic acids: gallic acid (*R*_t_ = 3.566, peak area = 8.344%), chlorogenic acid (*R*_t_ 4.231, 28.872%), methyl gallate (*R*_t_ 5.531, 7.832%), syringic acid (*R*_t_ 6.333, 1.429%), coumaric acid (*R*_t_ 8.585, 2.518%), vanillin (*R*_t_ 8.939, 2.357%), ferulic acid (*R*_t_ 9.531, 0.975%), rosmarinic acid (*R*_t_ 11.639, 0.618%), and cinnamic acid (*R*_t_ 19.110). In addition, the following five flavonoids were identified: rutin (*R*_t_ 6.911, 4.713%), naringenin (*R*_t_ 10.458, 7.354%), daidzein (*R*_t_ 15.640, 0.452%), quercetin (*R*_t_ 17.497, 2.323%), and kaempferol (*R*_t_ 20.334, 2.116%).

**FIGURE 1:**
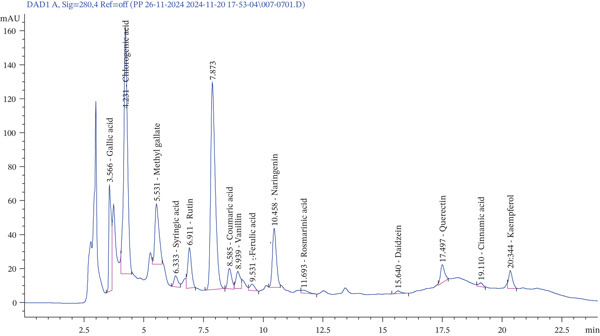
HPLC‐DAD chromatogram of the crude extract of *F. altissima* recorded at 280 nm. Compound identification was carried out by comparison of retention times with those of authentic reference standards analyzed under identical chromatographic conditions.

**FIGURE 2:**
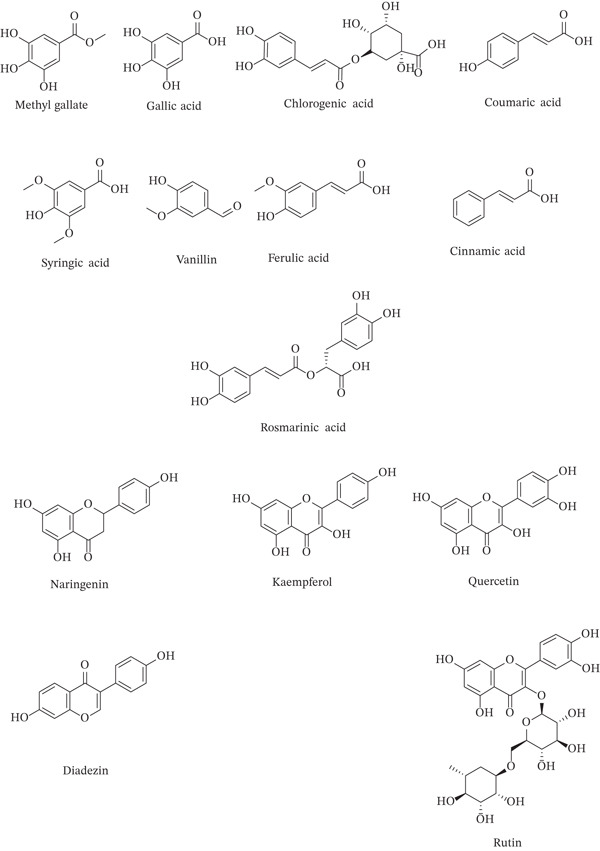
Chemical structure of the identified flavonoids and phenolic acids from crude extract of *F. altissima* using HPLC.

The chromatogram in Figure 1 and Table S2 illustrates the profile of these compounds, whereas the corresponding chemical structures of the detected flavonoids and phenolic acids are shown in Figure 2. Chlorogenic acid was the most detectable phenolic acid (28.872%). Gallic acid was 8.344%, and methyl gallate was 7.832%. Among flavonoids, naringenin was the most abundant compound (7.354%). Method validation confirmed the reliability of the results, providing a comprehensive chemical profile for *F. altissima*.

### 3.2. GC–MS Analysis of the Crude Extract

The crude extract of *F. altissima* was analyzed by GC–MS, and the total ion chromatogram (Table S3 and Figure S1) was analyzed based on retention times and spectral fragmentation patterns that were contrasted against reference values (Wiley and NIST Mass Spectral Library databases), which indicated the existence of several metabolites including seven fatty acids, three sugar derivatives (glucose, rhamnose, and xylose), and three phenolic acids (4‐hydroxy benzoic acid, vanillic acid, and protocatechuic acid). Additionally, the identified fatty acids included the following four unsaturated fatty acids: methyl‐10,11‐tetradecadienoate, methyl‐8,9‐octadecadienoate, methyl‐12,13‐tetradecadienoate, and alpha linoleic acid, as well as three fatty acids including stearic, myristic, and palmitic acids.

### 3.3. Quantification of Phenolic and Flavonoid Content of *F. altissima* Crude Extract

The pool of both phenolic and flavonoidal compounds in the extract was measured, and the values were calculated as means ± standard error (SE). The experiment revealed that the amount of total phenolic compounds was 144.81 ± 0.56 mg GAE/g, as calculated from the gallic acid (standard) calibration curve (Figure S2), reflecting a considerable concentration of phenolic compounds known for their antioxidant properties. Additionally, the total flavonoid content was 29.36 ± 0.19 mg QE/g as calculated from the quercetin (standard) calibration curve (Figure S3).

### 3.4. Antioxidant Activity of *F. altissima* Crude Extract

The free‐radical scavenging activity of *F. altissima* crude extract was evaluated using the DPPH assay. The extract exhibited a dose‐dependent inhibition with an IC_50_ value of 62.9 ± 1.9 *μ*g/mL, indicating a moderate antioxidant capacity (Figure S4). In comparison, the reference standard ascorbic acid showed significantly stronger activity with an IC_50_ value of 19.6 ± 2.4 *μ*g/mL (*p* < 0.05). These results demonstrate that although the extract possesses notable free‐radical scavenging ability, its antioxidant potency is approximately threefold lower than that of ascorbic acid (Figure S5).

### 3.5. Antimicrobial Activity of *F. altissima* Crude Extract

The antimicrobial activity of the *F. altissima* crude extract was assessed against six microbial strains by measuring the zone of inhibition (millimeters) as shown in Table 1. The extract demonstrated significant inhibition against four bacteria and one fungus stains compared to gentamicin (antibacterial) and fluconazole (antifungal) controls, respectively.

**TABLE 1:** Antimicrobial activity of *F. altissima* crude extract against selected microorganisms.

Microorganism	ZOI (FA extract)	ZOI (positive control)
*Bacillus subtilis* (ATCC 6633)	25 ± 0.2 mm	28 ± 0.2 mm
*Staphylococcus aureus* (ATCC 6538)	24 ± 0.2 mm	32 ± 0.1 mm
*Escherichia coli* (ATCC 8739)	16 ± 0.2 mm	24 ± 0.3 mm
*Klebsiella pneumoniae* (ATCC 13883)	15 ± 0.1 mm	22 ± 0.1 mm
*Candida albicans* (ATCC 10221)	18 ± 0.3 mm	30 ± 0.2 mm
*Aspergillus niger* (ATCC 16888)	No activity (NA)	32 ± 0.1 mm

*Note:* Data are expressed as mean ± SD; positive control against bacteria is gentamycin; positive control against fungus is fluconazole; zone of inhibition (ZOI) is classified into four grades; > 20 mm = very strong; 10–20 mm = strong, 5–10 mm = medium; and < 5 mm = no antimicrobial activity.

Abbreviation: FA extract = *F. altissima* extract.

As listed in Table 1, the crude extract of *F. altissima* showed moderate activity against four bacterial species *B. subtilis*, *S. aureus, E. coli*, and *K. pneumonia* with ZOI equal to 25 ± 0.2 mm, 24 ± 0.2 mm, 16 ± 0.2 mm, and 15 ± 0.1 mm, respectively. Also, *F. altissima* extract showed moderate activity against the fungal species *C. albicans* with ZOI equal to 18 ± 0.3 mm. However, it showed no activity against the fungal species *A. niger*.

The MIC of the crude extract of *F. altissima* was evaluated against selected four bacterial and one fungal strain. The extract exhibited strong antibacterial activity against *B. subtilis* and *S. aureus* with MIC values of 15.6 *μ*g/mL. In contrast, higher MIC values were observed against Gram‐negative strains, where *E. coli* and *K. pneumoniae* showed MICs of 62.5 *μ*g/mL, respectively. The antifungal activity against *C. albicans* also showed an MIC value of 31.2 *μ*g/mL.

### 3.6. Cytotoxic Activity

**FIGURE 3:**
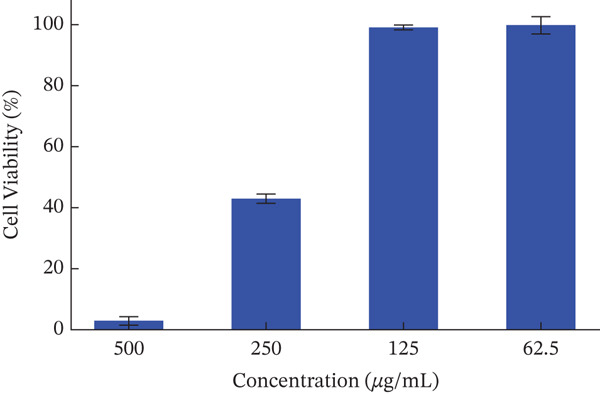
Cytotoxicity of the *F. altissima* extract using a range of concentrations (500, 250, 125, and 62.5) *μ*g/mL in RAW 264.7 cells, assessed using MTT colorimetric assay. The data are expressed as the mean ± standard deviation (SD) based on three replicates.

The crude extract of *F. altissima* did not express any significant cytotoxic activity below 100 *μ*g/mL (Figure 3) compared to untreated cells (considered 100% viability) using MTT colorimetric assay.

### 3.7. In Vitro Anti‐Inflammatory Activity of *F. altissima* Extract

The anti‐inflammatory properties of *F. altissima* crude extract were assessed by examining its effects on the gene expression levels of NF‐*κ*B and TNF‐*α* after LPS‐induced inflammation in RAW 264.7 cells. As illustrated in Table 2 and Figure S6, treatment of cells with LPS induced a significant increase in the expression of both inflammatory markers NF‐*κ*B and TNF‐*α* compared to control untreated cells. However, treating RAW cells which received LPS with the crude extract significantly downregulated the expression of both markers at a concentration of 100 *μ*g/mL compared to cells treated with LPS only.

**TABLE 2:** Effect of *F. altissima* crude extract on gene expression of TNF‐*α* and NF‐*κ*B in LPS‐treated RAW 264.7 cells.

Group	TNF‐*α* relative gene expression ± SD	NF‐*κ*B relative gene expression ± SD
Control	1.01 ± 0.3 ^∗^	1.0 ± 0.1 ^∗^
LPS (1 *μ*g/mL)	49.1 ± 8.2	43.7 ± 2.7
FA	4.43 ± 1.2 ^∗^	6.2 ± 0.09 ^∗^

*Note:* Data are presented as mean ± SD. Statistical significance was reported as *p* < 0.05.

Abbreviations: control = untreated RAW 264.7 cells; FA = RAW 264.7 cells treated with *F. altissima* 100 *μ*g/mL + LPS 1 *μ*g/mL in RAW 264.7; LPS = lipopolysaccharide‐treated RAW 264.7 cells (1 *μ*g/mL).

∗Indicates significance compared to the LPS group.

### 3.8. Topical Formulation

The crude extract of *F. altissima* was formulated into two preparations: hydrogel and ointment. Both formulations were found to be free of grittiness, homogeneous, and without phase separation. The pH of the formulations was 5.8 ± 0.1 for the FAHG preparation and 5.6 ± 0.25 for the *F. altissima* ointment (FAO). The pH values for both formulations fall within the normal skin pH range, making them less likely to produce skin irritation. Viscosity of FAHG and FAO was found to be acceptable and suitable for topical application, and recorded as 4090 ± 150 cP and 20,674 ± 200 cP, respectively. Additionally, the spreadability of the gel formulations was superior to that of the ointment (8.4 ± 0.3 g · cm/s vs. 2.3 ± 0.2 g · cm/s).

### 3.9. In Vivo Investigation

#### 3.9.1. Assessment of *F. altissima* Effect on Wound Healing

The effectiveness of the hydrogel (FAHG) and ointment (FAO) formulations of *F. altissima* extract in promoting wound healing was evaluated by measuring wound closure percentages over time in comparison to untreated group (negative control) and reference‐treated group (positive control). All treatment groups maintained stable body weight and showed no signs of toxicity, indicating no adverse effects from the applied formulations. Wound closure progressed in all groups over time, with reference‐treated group and FAO‐treated group demonstrating a significantly higher percentage of wound closure after 3 days compared to untreated controls. On Day 6, all three groups exhibited significant wound closure compared to untreated groups.

**FIGURE 4:**
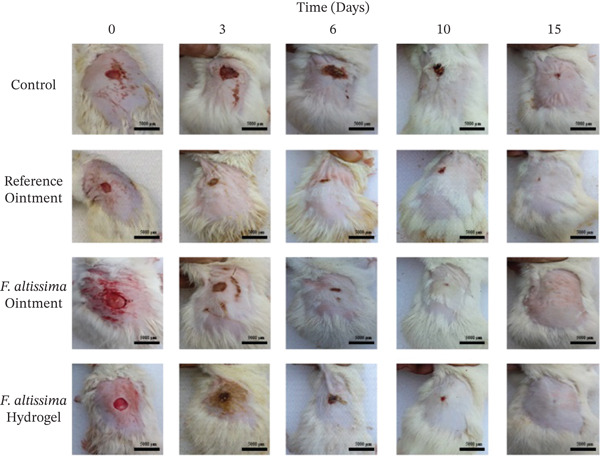
Representative images of wound closure in rats treated with different formulations over a 15‐day period. Full‐thickness excisional wounds were created and were either untreated (Control) or treated with a reference ointment, *F. altissima* ointment, or *F. altissima* hydrogel. Images were captured at the same distance on Days 0, 3, 6, 10, and 15 postwounding. Each image is representative of six replicates (*n* = 6). All scale bars are 5000 *μ*m.

**FIGURE 5:**
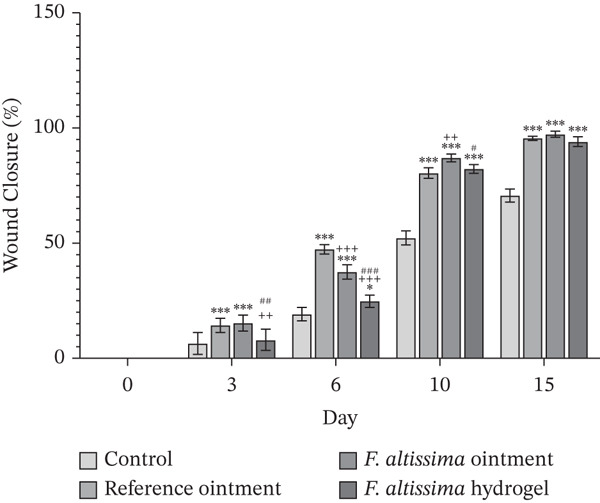
Time‐course analysis of wound closure percentage in experimental groups. The percentage of wound closure in the different treated groups was presented as mean ± SD (*n* = 6). The significance level (*α*) was set at 0.05. Statistical significance was reported as *p* < 0.05, *p* < 0.01, and *p* < 0.001, as ∗ indicates significance compared to the negative control group, + indicates significance compared to reference‐treated group, and # indicates significance compared to *F. altissima* ointment‐treated group.

By Day 10, all treated groups retained their significantly higher wound closure percentages compared to untreated controls. It was also noted that the FAO‐treated group showed significantly higher closure percentage compared to reference and FAHG groups. The treated groups remained significantly superior to untreated control at the 15. Notably, after 15 days there were no significant differences among the three treated groups regarding the percentage of wound closure as shown in Figures 4 and 5. By Day 21, complete wound closure (100%) was observed in all groups except for the untreated control group. It should be noted that wounds in the negative control group exhibited mild inflammation and incomplete closure after 15 days (Figure 4).

#### 3.9.2. Histopathological Evaluation of Wound Healing

**FIGURE 6:**
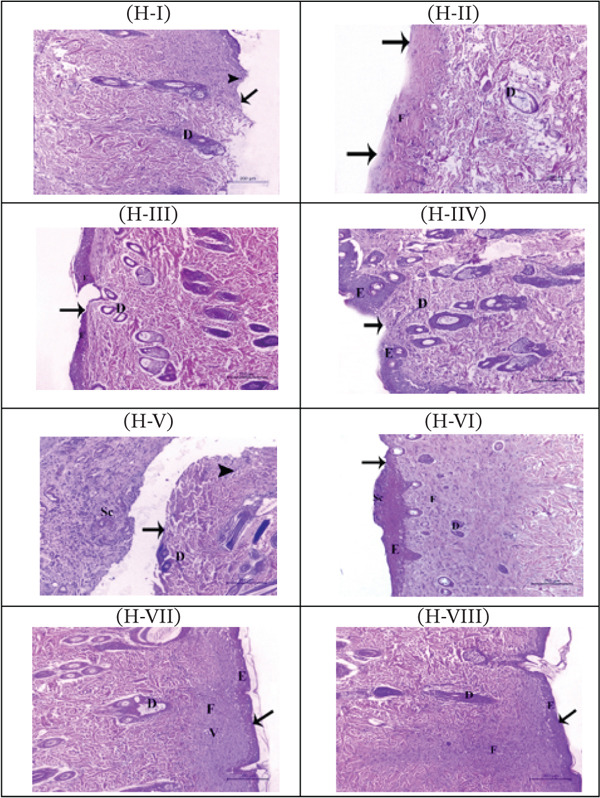
Photomicrographs for H&E staining of the skin wounded area from the different test groups after 2 days (upper panel H‐I to H‐IV) and 21 days (lower panel H‐V to H‐VIII) of wound incision. H‐I to H‐IV: sections from the control group (H‐I) indicating severe damage in the epidermal (E) and the dermis with denuded skin with no epithelium (arrow), severe inflammatory response and inflammatory cell infiltration (arrowhead). Reference‐treated group (H‐II) showing partial epithelization (arrow) of the wound area with underlying dermal tissue (D) with massive fibrosis (F). FAO‐treated group (H‐III) showing partial epithelization of the wound area with thin epithelial membrane (arrowhead) and normal underlying dermal structures. FAHG‐treated group (H‐IV) showing partial epithelization of the wound area with thin epithelial membrane (arrowhead). Hematoxylin and eosin staining, scale bar = 100 *μ*m, 200×. Sections from control group (H‐V) showing severe destruction in the epidermal (E) and dermal layers with total loss of the epithelial covering (arrow). Severe inflammatory response in the affected area with marked scar tissue (Sc) and severe inflammatory cell accumulation (arrowhead). Reference‐treated group (H‐VI) indicating full reepithelization (arrow) and it was covered by some scar tissue (Sc) with underlying normal dermal tissue (D) with marked fibrosis in the wound area (F). FAO‐treated group (H‐V‐II) indicating full reepithelization with epithelial membrane (arrow) and normal underlying dermal tissue (D). FAHG‐treated group (H‐VIII) indicating full reepithelization with thick epithelial membrane (arrow) and normal underlying dermal tissue (D).

Histological investigation of skin sections collected 2 days and 21 days postwounding from the different test groups showed that the control group exhibited severe structural disruptions, characterized by complete loss of the epithelial layer, extensive destruction of the epidermis and dermis, marked edema, and evident inflammatory cell infiltration after 2 days (Figure 6; H‐I). Some of these pathological changes were still visible after 21 days, such as damaged epidermis, edema, and infiltrating cells in addition to scar tissue formation (Figure 6; H‐V). In contrast, the reference ointment treatment resulted in partial re‐epithelialization with the formation of a thin epithelial layer over the wound area, accompanied by inflammatory cell infiltration and edema in the underlying dermal tissue 2 days after injury (Figure 6; H‐II). Twenty‐one days later, the wound site showed marked signs of healing with minimal scar tissue formation (Figure 6; H‐VI). Skin sections from rats treated with the FAO‐treated animals showed partial re‐epithelialization with a well‐organized but relatively thin epithelial layer, along with restored dermal architecture and minimal inflammatory response as early as 2 days after wounding (Figure 6; H‐III). Interestingly, skin sections from FAHG‐treated rats at the same time point exhibited a better skin architecture, as evidenced by complete re‐epithelialization with a thicker epithelial layer and fully restored dermal tissue, indicating enhanced wound closure and tissue remodeling compared to other groups (Figure 6; H‐IV). After 21 days of wound induction, sections from both FAO and FAHG‐treated groups showed complete healing with complete epithelization of the wound area, an intact epithelial membrane, and normal underlying dermal tissue (Figure 6; H‐VII and VIII).

#### 3.9.3. Immunohistological Assessment of VEGF Expression

**FIGURE 7:**
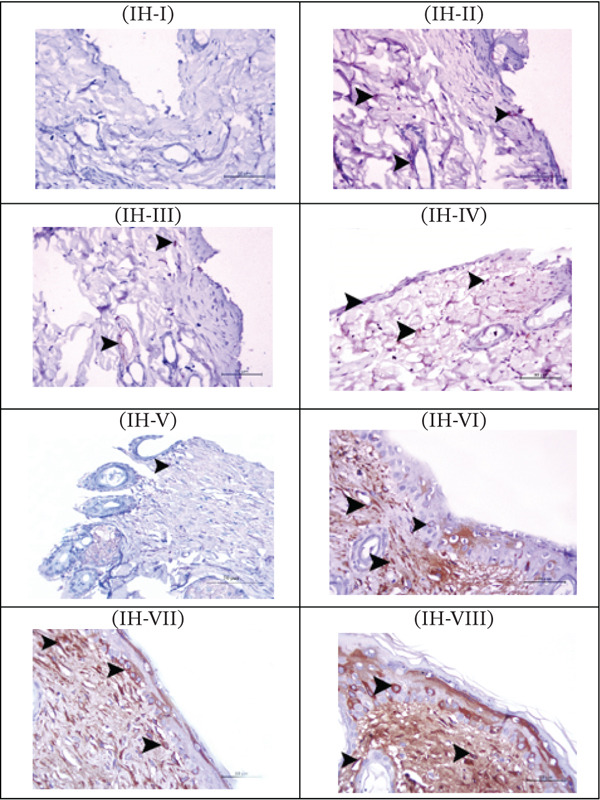
Expression of VEGF in the wound area in rats under different treatments after 2 days (IH‐I IH‐IV) and 21 days (IH‐V to IH‐VIII) of wound incision. IH‐I and IH‐V: sections from the untreated control group, showing no signal for VEGF in the wound area after 2 days, and a faint positive immune reaction in blood vessel endothelium (arrow) after 21 days. Sections from reference‐treated wounds showing positive immune reaction in the fibroblast (arrowhead) in the wound area after 2 days (IH‐II), which developed into massive immune reaction in the in the same cell types within the healed area (IH‐VI). Sections from the FAO‐treated group showing positive immune reaction in blood vessel endothelium and in the fibroblast (arrowhead) in the wound area after 2 days (IH‐III), which markedly increased by day 21 (IH‐VII). Sections from the FAHG‐treated group showing positive immune reaction in blood vessel endothelium and fibroblast (arrowhead) in the wound area after 2 days (IH‐IV) and the signal progressively increased by time (IH‐VIII). (VEGF immunostained 200×, scale bars = 100 *μ*m).

Skin sections from the wound area of the different test groups collected 2 and 21 days postinjury were immunostained for VEGF as a marker for revascularization. Sections collected from the control group showed almost a negative signal for VEGF after 2 days **(**Figure 7 **; IH-I)**; however, such tissues revealed a positive signal for VEGF in fibroblasts and endothelial cells of the blood vessels (arrowheads, Figure 7; IH‐V). When wounds were treated with the reference ointment, a positive signal for VEGF was detected in the fibroblasts after 2 days of injury (arrowhead, Figure 7; IH‐II). By time, the VEGF expression signal in fibroblasts (arrowhead) became stronger, and it was also detectable in vascular epithelium (Figure 7; IH‐VI). It is to be noted that treating the wound area with either FA ointment or hydrogel resulted in a detectable signal for VEGF in fibroblasts and endothelial lining of blood vessels within the wound area 2 days after wound induction (arrowheads Figure 7; H‐III and H‐IV). This expression of VEGF developed to show a strong, widely distributed signal in fibroblasts and endothelial lining of blood vessels within the wound area after 21 days of treatment with FAO or FAHG (arrowheads, Figure 7; H‐VII and H‐VIII).

## 4. Discussion

Wound healing is a highly coordinated and multistage biological process involving inflammation, proliferation, and tissue remodeling through the interaction of various cellular and molecular components. This complex continuous step ultimately leads to restoration of tissue integrity and function ([1, 2]; Madea & Pollak, 2014). Recently, natural products have gained considerable attention as potential alternatives to synthetic drugs due to their lower cost, reduced side effects, and higher efficacy based on the presence of multiple bioactive constituents that can act synergistically.

In the present study, direct macroscopic observation of excision wound models revealed that wounds treated with FAHG and FAO exhibited faster and more complete healing compared to the positive control group. The enhanced wound contraction and re‐epithelialization observed by day 21 suggest that *F. altissima* extracts actively promoted the progression from the inflammatory phase to the proliferative and remodeling phases of wound repair. These findings were further supported by histopathological examinations, which demonstrated an intact epithelial layer and well‐organized dermal tissue in both treated groups. Such histological features indicate improved collagen deposition, fibroblast proliferation, and tissue regeneration, which are essential hallmarks of effective wound healing. Immunohistochemical analysis revealed increased VEGF expression in fibroblasts of *F. altissima*‐treated groups. VEGF is a critical regulator of angiogenesis during the proliferative phase of wound healing, facilitating neovascularization and improving oxygen and nutrient supply to regenerating tissues. Enhanced VEGF expression suggests that *F. altissima* extract may stimulate angiogenic pathways, thereby accelerating granulation tissue formation and epithelial regeneration. Although VEGF is a key marker of angiogenesis, wound healing is regulated by multiple growth factors and cytokines.

Additionally, microbial infection is one of the major factors that delay wound healing by prolonging the inflammatory phase and causing tissue destruction. Pathogenic bacteria such as *K. pneumoniae*, *E. coli*, and *S. aureus* are frequently associated with wound infections and are capable of forming biofilms that enhance their resistance to treatment [39]. The moderate antibacterial activity observed for the crude extract of *F. altissima* against these pathogens indicates that the extract may help reduce microbial burden at the wound site, thereby preventing secondary infections and facilitating faster tissue repair. This antimicrobial property likely contributes indirectly to the improved wound healing observed in vivo by minimizing persistent inflammation and cellular damage caused by bacterial colonization.

Furthermore, the extract of *F. altissima* demonstrated significant inhibition of inflammatory markers TNF‐*α* and NF‐*κ*B. These mediators play central roles in the initiation and maintenance of inflammatory responses. Excessive or prolonged activation of TNF‐*α* and NF‐*κ*B can impair wound healing by inducing oxidative stress and delaying cell proliferation. Therefore, suppression of these pathways suggests that *F. altissima* extract may modulate the inflammatory phase of wound healing, promoting a timely transition to the proliferative stage. In addition, the antioxidant activity of the extract, reflected by its free‐radical scavenging capacity (IC_50_ = 62.9 ± 1.9 *μ*g/mL), may further protect wound tissues from oxidative damage and enhance cellular survival and repair processes.

Phytochemical analysis of *F. altissima* extract by HPLC and GC–MS revealed the presence of polyphenols, flavonoids, and polyunsaturated fatty acids, which are well known for their pharmacological activities. Chlorogenic acid, gallic acid, methyl gallate and naringenin, and protocatechuic acid were identified as major constituents and may play key roles in the observed biological effects. Chlorogenic acid promotes healing by modulating TGF‐*β*1 and TNF‐*α* factors, reducing oxidative stress, and increasing fibroblast migration and collagen synthesis [40]. Additionally, protocatechuic acid, gallic acid, and methyl gallate showed a potent free‐radical scavenging activity which accelerated wound healing and prevented contaminations [41, 42].

Moreover, flavonoids and polyphenols have been reported to promote wound healing through multiple mechanisms, including antimicrobial, antioxidant activity, inhibition of pro‐inflammatory cytokines, stimulation of fibroblast migration, enhancement of collagen synthesis, and promotion of angiogenesis [43, 44]. Moreover, unsaturated fatty acids have attracted increasing interest in wound care due to their ability to maintain cell membrane integrity, regulate inflammatory responses, and support tissue regeneration [45].

Previous studies have demonstrated the wound‐healing potential of different *Ficus* species formulated as ointments or hydrogels [23, 46, 47]. The findings of the present study are in agreement with these reports and further extend the therapeutic relevance of the genus *Ficus* by highlighting the bioactivity of *F. altissima*. Compared with earlier studies, the current work provides combined evidence of antimicrobial, anti‐inflammatory, antioxidant, and angiogenic activities, suggesting that these effects may act synergistically to accelerate wound closure and tissue regeneration.

Overall, the present results indicate that *F. altissima* possesses significant wound‐healing potential mediated through modulation of inflammation, enhancement of angiogenesis, reduction of microbial load, and protection against oxidative stress. These multiple mechanisms likely operate together to promote faster and more organized tissue repair. However, further studies are required to isolate and evaluate individual active constituents, elucidate precise molecular pathways, and confirm safety and efficacy through long‐term and clinical investigations.

## 5. Study Limitations and Prospects

While the present study provides valuable evidence supporting the wound‐healing potential of *F. altissima* extract in vitro and in vivo, some aspects warrant further consideration. The investigation was performed using a crude extract, and therefore the contribution of individual bioactive constituents could not be fully distinguished. Moreover, the experimental period and animal model applied offer an initial assessment of efficacy, but additional studies including longer observation times and broader safety evaluations would help to further strengthen and validate these findings.

Our ongoing research focuses on the bio‐guided isolation of metabolites responsible for the extract′s activity, along with evaluating its antifungal properties against various fungal species commonly found in wound infections. Additionally, it is interesting to investigate how the compounds in the *F. altissima* extract interact synergistically to enhance their effects. Understanding these mechanisms will enable the development of optimized formulations for specific clinical conditions, ultimately leading to pharmaceutical products tailored to address medical needs.

## 6. Conclusions

This study explored the pharmacological potential and phytochemical characterization of *F. altissima* aerial parts. A key contribution of this work is the first‐time documentation of their phytochemical profile together with demonstrated wound‐healing, anti‐inflammatory, antimicrobial, and antioxidant activities. These findings highlight the therapeutic promise of *F. altissima* as a natural source of bioactive compounds. Future studies should focus on the isolation and characterization of the major active constituents, elucidation of their molecular mechanisms of action, and comprehensive safety and toxicity evaluations.

**Nomenclature**
HPLC: high‐performance liquid chromatography
GC–MS: gas chromatography–mass spectrometry
NF‐*κ*B: nuclear factor‐kappa B
TNF‐*α*
: tumor necrosis factor‐alpha
RAW: macrophage cell line
LSP: lipopolysaccharides
RT‐qPCR: quantitative reverse transcription polymerase chain reaction
VEGF: vascular endothelial growth factor

## Author Contributions

Conceptualization: Hossein M. Elbadawy, Hamad Alrbyawi, and Fahd M. Abdelkarem; methodology: Aryam S. Alharbi, Asalah B. Alhazmi, Manhal N. Hudhayri, Salma A. Ibrahim, Maya A. Alhazmi, and Shuruq M. Almohammadi, and Mekky M. Abouzied; validation: Hossein M. Elbadawy, Ahmed Aldhafiri, and Heba M. Eltahir; formal analysis: Mekky M. Abouzied, Mohamed R. Kamel, and Hossein M. Elbadawy, and Hamad Alrbyawi; investigation: Hamad Alrbyawi, Hossein M. Elbadawy, Fahd M. Abdelkarem, and Mohamed R. Kamel; data curation: Hossein M. Elbadawy and Mekky M. Abouzied; writing—original draft preparation: Hossein M. Elbadawy, Hamad Alrbyawi, Aryam S. Alharbi, Asalah B. Alhazmi, Manhal N. Hudhayri, Salma A. Ibrahim, Maya A. Alhazmi, Shuruq M. Almohammadi, and Mekky M. Abouzied; writing—review and editing: Heba M. Eltahir, Hossein M. Elbadawy, Mekky M. Abouzied, and Hamad Alrbyaw; visualization: Fahd M. Abdelkarem, Hossein M. Elbadawy, Hamad Alrbyawi, and Mohamed R. Kamel; supervision: Hossein M. Elbadawy, Hamad Alrbyawi, and Heba M. Eltahir; project administration: Hossein M. Elbadawy and Hamad Alrbyawi.

## Funding

No funding was received for this manuscript.

## Disclosure

All authors have read and agreed to the published version of the manuscript.

## Ethics Statement

The study was conducted at the Faculty of Pharmacy, Taibah University, KSA, and approved by the Research Ethics Committee (COPTU‐REC) with reference number COPTU‐REC‐127‐20251002 on 10/02/2025.

## Consent

The authors have nothing to report.

## Conflicts of Interest

The authors declare no conflicts of interest.

## Supporting information


**Supporting Information** Additional supporting information can be found online in the Supporting Information section. Table S1: Primer Sequences for TNF‐*α*, NF‐*κ*B and Gapdh. Table S2: List of identified phenolic acids and flavonoids from the crude extract of Ficus altissima using HPLC by comparing with authentic standards. Table S3: List of bioactive compounds identified from the crude extract of Ficus altissima using Gas Chromatography–Mass Spectrometry (GC–MS). Figure S1: GC–MS total ion chromatogram (TIC) of the crude extract of Ficus altissima. Peaks are numbered according to their order of elution. Compound identification was performed by comparison of mass spectra, fragmentation pattern with those in the NIST library. Figure S2: Calibration curve of gallic acid for the determination of total phenolic content (TPC) in the crude extract of *Ficus altissima*. Figure S3: Calibration curve of quercetin (*μ*g/mL) for the determination of total flavonoid content (TFC) in the crude extract of *Ficus altissima*. Figure S4: Antioxidant activity of Ficus altissima extract evaluated by the DPPH radical scavenging assay at different concentrations (6.25–100 *μ*g/mL). Data are expressed as percentage of DPPH inhibition (mean ± SD, n = 3). Figure S5: Antioxidant activity of ascorbic acid evaluated by the DPPH radical scavenging assay at different concentrations (6.25–100 *μ*g/mL). Data are expressed as percentage of DPPH inhibition (mean ± SD, n = 3). Figure S6: Effect of F. altissima crude extract on gene expression of TNF‐*α* and NF‐*κ*B in LPS‐treated RAW 264.7 cells. Control = untreated RAW 264.7 cells, LPS = Lipopolysaccharide‐treated RAW 264.7 cells (1 *μ*g/mL); FA = RAW 264.7 cells treated with F. altissima 100 *μ*g/mL + LPS 1 *μ*g/mL in RAW 264.7. data is presented as mean ± SD. Statistical significance was reported as p <0.05, ∗ indicates significance compared to the LPS group.

## Data Availability

Data supporting the findings of this study are available in the supporting information associated with this article.

## References

[1] Kirwan H. O. and Pignataro R. , The Skin and Wound Healing, Pathology and Intervention in Musculoskeletal Rehabilitation. (2015) 25, no. 8, 1352–1356.

[2] Shukla S. K. , Sharma A. K. , Gupta V. , and Yashavarddhan M. H. , Pharmacological Control of Inflammation in Wound Healing, Journal of Tissue Viability. (2019) 28, no. 4, 218–222, 10.1016/j.jtv.2019.09.002, 2-s2.0-85072247208. 31542301

[3] Madea B. , Pollak S. , Thierauf A. , Meissner C. , Oehmichen M. , and Leth P. M. , Tsokos M. , Mechanical Trauma and Classification of Wounds, Handbook of Forensic Medicine. (2014) Springer, 253–327.

[4] Singh S. , Young A. , and McNaught C. E. , The Physiology of Wound Healing, Surgery. (2017) 35, no. 9, 473–477, 10.1016/j.mpsur.2017.06.004, 2-s2.0-85026242858.

[5] Falcone M. , De Angelis B. , Pea F. , Scalise A. , Stefani S. , Tasinato R. , Zanetti O. , and Dalla Paola L. , Challenges in the Management of Chronic Wound Infections, Journal of Global Antimicrobial Resistance. (2021) 26, 140–147, 10.1016/j.jgar.2021.05.010. 34144200

[6] Tam J. C. , Lau K. M. , Liu C. L. , To M. H. , Kwok H. F. , Lai K. K. , Lau C. P. , Ko C. H. , Leung P. C. , Fung K. P. , and Lau C. B. , The *In Vivo* and *In Vitro* Diabetic Wound Healing Effects of a 2-Herb Formula and Its Mechanisms of Action, Journal of Ethnopharmacology. (2011) 134, no. 3, 831–838, 10.1016/j.jep.2011.01.032, 2-s2.0-79954415660. 21291991

[7] Rizvi S. A. , Einstein G. P. , Tulp O. L. , Sainvil F. , and Branly R. , Introduction to Traditional Medicine and Their Role in Prevention and Treatment of Emerging and Re-Emerging Diseases, Biomolecules. (2022) 12, no. 10, 10.3390/biom12101442. PMC9599697 36291651

[8] Schilrreff P. and Alexiev U. , Chronic Inflammation in Wound Healing: The Role of Natural Products, International Journal of Molecular Sciences. (2022) 23, no. 9, 10.3390/ijms23094928. PMC9104327 35563319

[9] Chaachouay N. and Zidane L. , Plant-Derived Natural Products: A Source for Drug Discovery and Development, Drugs and Drug Candidates. (2024) 3, no. 1, 184–207, 10.3390/ddc3010011.

[10] Cedillo-Cortezano M. , Martinez-Cuevas L. R. , López J. A. M. , Barrera López I. L. , Escutia-Perez S. , and Petricevich V. L. , Use of Medicinal Plants in the Process of Wound Healing: A Literature Review, Pharmaceuticals. (2024) 17, no. 3, 10.3390/ph17030303. PMC10975678 38543089

[11] Agyare C. , Boakye Y. D. , Bekoe E. O. , Hensel A. , Dapaah S. O. , and Appiah T. , African Medicinal Plants With Wound Healing Properties, Journal of Ethnopharmacology. (2016) 177, 85–100, 10.1016/j.jep.2015.11.008, 2-s2.0-84948798825. 26549271

[12] Ghosh P. K. and Gaba A. , Phyto-Extracts in Wound Healing, Journal of Pharmacy and Pharmaceutical Sciences. (2013) 16, no. 5, 760–820, 10.18433/J3831V. 24393557

[13] Kolimi P. , Narala S. , Nyavanandi D. , Youssef A. A. A. , and Dudhipala N. , Innovative Treatment Strategies to Accelerate Wound Healing: Trajectory and Recent Advancements, Cells. (2022) 11, no. 15, 10.3390/cells11152439. PMC9367945 35954282

[14] Ramadan M. A. , Ahmad A. S. , Nafady A. M. , and Mansour A. I. , Chemical Composition of the Stem Bark and Leaves of *Ficus pandurata* Hance, Natural Product Research. (2009) 23, no. 13, 1218–1230, 10.1080/14786410902757899, 2-s2.0-71049177472. 19731141

[15] Zeng Y. , Ye W. , Huang J. , Li C. , and Giblin-Davis R. M. , Description of *Schistonchus altissimus* n. sp. (Nematoda: Aphelenchoididae), an Associate of *Ficus altissima* in China, Zootaxa. (2013) 3700, no. 4, 561–572. 26106743 10.11646/zootaxa.3700.4.4

[16] Sieniawska E. , Świątek Ł. , Sinan K. I. , Zengin G. , Boguszewska A. , Polz-Dacewicz M. , Bibi Sadeer N. , Etienne O. K. , and Mahomoodally M. F. , Phytochemical Insights Into *Ficus sur* Extracts and Their Biological Activity, Molecules. (2022) 27, no. 6, 10.3390/molecules27061863. PMC8949149 35335228

[17] Salem M. Z. , Salem A. Z. M. , Camacho L. M. , and Ali H. M. , Antimicrobial Activities and Phytochemical Composition of Extracts of *Ficus* Species: An Overview, African Journal of Microbiology Research. (2013) 7, no. 33, 4207–4219, 10.5897/AJMR2013.5570.

[18] Walia A. , Kumar N. , Singh R. , Kumar H. , Kumar V. , Kaushik R. , and Kumar A. P. , Bioactive Compounds in *Ficus* fruits, Their Bioactivities, and Associated Health Benefits: A Review, Journal of Food Quality. (2022) 2022, no. 1, 6597092, 10.1155/2022/6597092.

[19] Zhang D. L. , Feng Y. H. , Liang Z. Y. , Lin Q. , and Xu J. , Chemical Composition of Essential Oil From Fruit of *Ficus altissima* , Advanced Materials Research. (2012) 554, 1125–1128, 10.4028/www.scientific.net/amr.554-556.1125, 2-s2.0-84867174558.

[20] Yao J. , Wang Z. , Wang R. , Wang Y. , Xu J. , and He X. , Anti-Proliferative and Anti-Inflammatory Prenylated Isoflavones and Coumaronochromones From the Fruits of *Ficus altissima* , Bioorganic Chemistry. (2021) 113, 104996, 10.1016/j.bioorg.2021.104996. 34038794

[21] Deng Y. T. , Liang G. , Shi Y. , Li H. L. , Zhang J. , Mao X. M. , Fu Q. R. , Peng W. X. , Chen Q. X. , and Shen D. Y. , Condensed Tannins From *Ficus altissima* Leaves: Structural, Antioxidant, and Antityrosinase Properties, Process Biochemistry. (2016) 51, no. 8, 1092–1099, 10.1016/j.procbio.2016.04.022, 2-s2.0-84992307230.

[22] Hwisa N. T. , Chandu B. R. , Katakam P. , and Nama S. , Pharmacognostical Studies on the Leaves of *Ficus altissima* Blume, Journal of Applied Pharmaceutical Science. (2013) 3, no. 4, S56–S58, 10.7324/JAPS.2013.34.S10, 2-s2.0-84880594553.

[23] Hashad I. M. , Aly S. H. , Saleh D. O. , Abo El-Nasr N. M. , Shabana M. E. , El-Tokhy F. S. , El-Nashar H. A. , Abdelmohsen U. R. , Mostafa N. M. , and Mostafa A. M. , Mechanistic Wound Healing of *Ficus trijuja* Leaf Extract and Its Lipid Nanocapsule Supported by Metabolomic Profiling and *In Vivo* Studies, International Journal of Molecular Sciences. (2025) 26, no. 3, 10.3390/ijms26030928. PMC11817089 39940701

[24] Singh B. , Sharma H. K. , and Sarkar B. C. , Optimization of Extraction of Antioxidants From Wheat Bran (*Triticum* spp.) Using Response Surface Methodology, Journal of Food Science and Technology. (2012) 49, no. 3, 294–308, 10.1007/s13197-011-0276-5, 2-s2.0-84863984211. 23729849 PMC3614047

[25] Aljohani A. K. B. , Alharbi A. S. , Alhazmi A. B. , Hudhayri M. N. , Almuwallad I. B. , Alhazmi M. A. , Almohammadi S. M. , Alsaleh A. I. , Aldhafiri A. , Eltahir H. M. , Abouzied M. M. , Alrbyawi H. , Mohamed M. S. , Abdel-Emam M. M. , and Abdelkarem F. M. , Antidiabetic Potential of Sea Urchin Tripneustes gratilla Nanosuspension Based on In Vitro Enzyme Inhibition, In Vivo Evaluation, and Chemical Profiling Approaches, Current Issues in Molecular Biology. (2025) 48, no. 1, 10.3390/cimb48010008. PMC12839668 41614839

[26] Aljohani A. K. , Maghrabi N. A. , Alrehili O. M. , Alharbi A. S. , Alsihli R. S. , Alharthe A. M. , Albladi R. S. , Alosaimi K. A. , Albadrani B. M. , Miski S. F. , Elbadawy H. M. , Alrehaili B. D. , Abdelkarem F. A. , and Hussein M. F. , Ajwa Date Extract (*Phoenix dactylifera* L.): Phytochemical Analysis, Antiviral Activity Against HSV-1 and Coxsackie B4 Virus, and In Silico Study, Saudi Medical Journal. (2025) 46, no. 1, 10.15537/smj.2025.46.1.20240780. PMC11717108 39779363

[27] Lamuela-Raventós R. M. , Apak R. , Capanoglu E. , and Shahidi F. , Folin–Ciocalteu Method for the Measurement of Total Phenolic Content and Antioxidant Capacity, Measurement of Antioxidant Activity & Capacity, 2018, Wiley, 107–115, 10.1002/9781119135388.ch6, 2-s2.0-85050756223.

[28] Chen H. H. and Zhou J. H. , Systematic Investigation on AlCl3 Colorimetric Determination of Total Flavonoids in Daylily, Proceedings of the International Conference on Biomedical and Intelligent Systems (IC-BIS 2022), 2022, Society of Photo-Optical Instrumentation Engineers, 12–21.

[29] Garcia E. J. , Oldoni T. L. C. , Alencar S. M. D. , Reis A. , Loguercio A. D. , and Grande R. H. M. , Antioxidant Activity by DPPH Assay of Potential Solutions to be Applied on Bleached Teeth, Brazilian Dental Journal. (2012) 23, 22–27, 10.1590/S0103-64402012000100004, 2-s2.0-84859135666. 22460310

[30] Erhonyota C. , Edo G. I. , and Onoharigho F. O. , Comparison of Poison Plate And Agar Well Diffusion Method Determining the Antifungal Activity of Protein Fractions, Acta Ecologica Sinica. (2023) 43, no. 4, 684–689, 10.1016/j.chnaes.2022.08.006.

[31] David W. W. and Stout T. R. , Disc Plate Method of Microbiological Antibiotic Assay. I. Factors Influencing Variability and Error, Applied Microbiology. (1971) 22, no. 4, 659–665, 10.1128/am.22.4.659-665.1971. 5002143 PMC376382

[32] Livak K. J. and Schmittgen T. D. , Analysis of Relative Gene Expression Data Using Real-Time Quantitative PCR and the 2−*ΔΔ*CT Method, Methods. (2001) 25, no. 4, 402–408, 10.1006/meth.2001.1262, 2-s2.0-0035710746. 11846609

[33] Lukić M. , Pantelić I. , and Savić S. D. , Towards Optimal pH of the Skin and Topical Formulations, Cosmetics. (2021) 8, no. 3, 10.3390/cosmetics8030069.

[34] Bayan M. F. , Chandrasekaran B. , and Alyami M. H. , Development and Characterization of Econazole Topical Gel, Gels. (2023) 9, no. 12, 10.3390/gels9120929. PMC10743284 38131915

[35] Suresh P. , Salem-Bekhit M. M. , Veedu H. P. , Alshehri S. , Nair S. C. , Bukhari S. I. , Viswanad V. , Taha E. I. , Sahu R. K. , Ghoneim M. M. , and Elbagory I. , Development of a Novel Methotrexate-Loaded Nanoemulsion for Rheumatoid Arthritis Treatment with Site-Specific Targeting Subcutaneous Delivery, Nanomaterials. (2022) 12, no. 8, 10.3390/nano12081299. PMC9027573 35458007

[36] Percie du Sert N. , Hurst V. , Ahluwalia A. , Alam S. , Avey M. T. , Baker M. , Browne W. J. , Clark A. , Cuthill I. C. , Dirnagl U. , Emerson M. , Garner P. , Holgate S. T. , Howells D. W. , Karp N. A. , Lazic S. E. , Lidster K. , MacCallum C. J. , Macleod M. , Pearl E. J. , Petersen O. H. , Rawle F. , Reynolds P. , Rooney K. , Sena E. S. , Silberberg S. D. , Steckler T. , and Würbel H. , The ARRIVE Guidelines 2.0: Updated Guidelines for Reporting Animal Research, Public Library of Science Biology. (2020) 18, no. 7, e3000410, 10.1371/journal.pbio.3000410. PMC7360023 32663219

[37] Zeng Q. , Xie H. , Song H. , Nie F. , Wang J. , Chen D. , and Wang F. , *In Vivo* Wound Healing Activity of *Abrus cantoniensis* Extract, Evidence-Based Complementary and Alternative Medicine. (2016) 2016, no. 1, 6568528, 10.1155/2016/6568528, 2-s2.0-85009359169. 28119760 PMC5227303

[38] van der Loos C. M. , Meijer-Jorna L. B. , Broekmans M. E. , Ploegmakers H. P. , Teeling P. , de Boer O. J. , and van der Wal A. C. , Anti-Human VEGF Antibody Selection for Immunohistochemical Staining, Journal of Histochemistry and Cytochemistry. (2010) 58, no. 2, 109–118, 10.1369/jhc.2009.954586, 2-s2.0-76649140511. 19786611 PMC2803701

[39] Liu S. , Long Q. , Xu Y. , Wang J. , Xu Z. , Wang L. , Zhou M. , Wu Y. , Chen T. , and Shaw C. , Assessment of Antimicrobial and Wound Healing Effects of Brevinin-2Ta Against *Klebsiella pneumoniae* in Dermally Wounded Rats, Oncotarget. (2017) 8, no. 67, 111369, 10.18632/oncotarget.22797, 2-s2.0-85038432218. 29340060 PMC5762328

[40] Nguyen V. , Taine E. G. , Meng D. , Cui T. , and Tan W. , Chlorogenic Acid: A Systematic Review on the Biological Functions, Mechanistic Actions, and Therapeutic Potentials, Nutrients. (2024) 16, no. 7, 10.3390/nu16070924. PMC11013850 38612964

[41] Elhewehy A. A. , El-Fishawy A. M. , Aly R. M. , Mohsen E. , and Fayed M. A. , Isolation and Quantification of Polyphenolics, Exploration of Antioxidant, Cytotoxicity, and Wound Healing Activities of *Pithecellobium dulce* (Roxb.) Benth, Scientific Reports. (2026) 16, no. 1, 10.1038/s41598-025-32257-7. PMC12795817 41521222

[42] Zhou C. , Xu R. , Han X. , Tong L. , Xiong L. , Liang J. , Sun Y. , Zhang X. , and Fan Y. , Protocatechuic Acid-Mediated Injectable Antioxidant Hydrogels Facilitate Wound Healing, Composites Part B: Engineering. (2023) 250, 110451, 10.1016/j.compositesb.2022.110451.

[43] Melguizo-Rodríguez L. , de Luna-Bertos E. , Ramos-Torrecillas J. , Illescas-Montesa R. , Costela-Ruiz V. J. , and García-Martínez O. , Potential Effects of Phenolic Compounds Found in Olive Oil on Wound Healing, Foods. (2021) 10, no. 7, 10.3390/foods10071642. PMC8307686 34359512

[44] Zulkefli N. , Che Zahari C. N. M. , Sayuti N. H. , Kamarudin A. A. , Saad N. , Hamezah H. S. , Bunawan H. , Baharum S. N. , Mediani A. , Ahmed Q. U. , Ismail A. F. H. , and Sarian M. N. , Flavonoids as Potential Wound-Healing Molecules: Emphasis on Pathways Perspective, International Journal of Molecular Sciences. (2023) 24, no. 5, 10.3390/ijms24054607. PMC10003005 36902038

[45] Hokynková A. , Nováková M. , Babula P. , Sedláčková M. , Paulová H. , Hlaváčová M. , Charwátová D. , and Stračina T. , Fatty Acid Supplementation Affects Skin Wound Healing in a Rat Model, Nutrients. (2022) 14, no. 11, 10.3390/nu14112245. PMC9182784 35684045

[46] Arunachalam K. and Parimelazhagan T. , Anti-Inflammatory, Wound Healing and *In Vivo* Antioxidant Properties of the Leaves of *Ficus amplissima* Smith, Journal of Ethnopharmacology. (2013) 145, no. 1, 139–145, 10.1016/j.jep.2012.10.041, 2-s2.0-84871274005. 23123798

[47] Nayeem N. , Rohini R. , Asdaq S. M. , and Das A. , Wound Healing Activity of the Hydroalcoholic Extract of *Ficus religiosa* Leaves in Rats, Internet Journal of Alternative Medicine. (2009) 6, 1–5.

