# Supplementary material for: Phytochemical Analysis, In Vitro and In Vivo Evaluation of Ficus altissima Extract‐Based Ointment and Hydrogel on Wound Healing
Source: Biomed Res Int. 2026 Apr 28;2026:5137800. doi: 10.1155/bmri/5137800 (PMC13122729; doi:10.1155/bmri/5137800)
Supplement: Supplementary file 1 — Supporting Information Additional supporting information can be found online in the Supporting Information section. Table S1: Primer Sequences for TNF‐α, NF‐κB and Gapdh. Table S2: List of identified phenolic acids and flavonoids from the crude extract of Ficus altissima using HPLC by comparing with authentic standards. Table S3: List of bioactive compounds identified from the crude extract of Ficus altissima using Gas Chromatography–Mass Spectrometry (GC–MS). Figure S1: GC–MS total ion chromatogram (TIC) of the crude extract of Ficus altissima. Peaks are numbered according to their order of elution. Compound identification was performed by comparison of mass spectra, fragmentation pattern with those in the NIST library. Figure S2: Calibration curve of gallic acid for the determination of total phenolic content (TPC) in the crude extract of Ficus altissima. Figure S3: Calibration curve of quercetin (μg/mL) for the determination of total flavonoid content (TFC) in the crude extract of Ficus altissima. Figure S4: Antioxidant activity of Ficus altissima extract evaluated by the DPPH radical scavenging assay at different concentrations (6.25–100 μg/mL). Data are expressed as percentage of DPPH inhibition (mean ± SD, n = 3). Figure S5: Antioxidant activity of ascorbic acid evaluated by the DPPH radical scavenging assay at different concentrations (6.25–100 μg/mL). Data are expressed as percentage of DPPH inhibition (mean ± SD, n = 3). Figure S6: Effect of F. altissima crude extract on gene expression of TNF‐α and NF‐κB in LPS‐treated RAW 264.7 cells. Control = untreated RAW 264.7 cells, LPS = Lipopolysaccharide‐treated RAW 264.7 cells (1 μg/mL); FA = RAW 264.7 cells treated with F. altissima 100 μg/mL + LPS 1 μg/mL in RAW 264.7. data is presented as mean ± SD. Statistical significance was reported as p <0.05, ∗ indicates significance compared to the LPS group. [file BMRI-2026-5137800-s001.docx]

**Phytochemical Analysis, *In-Vitro* and *In-Vivo* Evaluation of *Ficus altissima* Extract Based-Ointment and Hydrogel on Wound Healing**

**Abstract:**

Objective: Wound healing is a sequential mechanism that occurs in four successive stages: hemostasis, inflammation, proliferation, and remodeling. Delayed wound healing and the subsequent complications can be observed in poorly controlled chronic diseases and immunocompromised patients. This study focused on the wound healing activity of Ficus altissima extract-based formulations, exploring their phytochemical and biological properties. Methods: Phytochemical characterization was conducted using HPLC, GC-MS, and spectroscopic methods for quantification of phenolic compounds and flavonoids. Disk diffusion was utilized to test the antimicrobial effects of the extract and antioxidant activity was measured using DPPH assay. Anti-inflammatory activity was assessed by the quantification of NF-κB and TNF-α levels in LPS-stimulated RAW 264.7 cells. An ointment and Hydrogel were prepared from F. altissima aerial parts and tested for their wound healing effect using skin wound healing model in rats. Results: Phenolic acids, flavonoid compounds, fatty acids, and sugar derivatives were detected in F. altissima extract with total phenolic and total flavonoid contents equal to 144.81 mg. GAE g-1 and 29.36 mg. QE g-1, respectively. F. altissima downregulated TNF-α and NF-κB expression and inhibited the microbial growth of various microorganisms. Both formulations improved wound closure, with the ointment showing superior results within 10 days. Conclusions: F. altissima extract, particularly in an ointment form, effectively promoted wound healing and tissue regeneration and this effect can be linked to the detected phenolic acids and flavonoids with anti-inflammatory, antioxidant and antimicrobial properties.

**Keywords:** *Ficus altissima*; Wounds healing; Phytochemical characterization; Anti-inflammatory; Ointment-based formulation

Table S1. Primer Sequences for TNF-α, NF-κB and Gapdh

| gene | forward primer | reverse primer |
| --- | --- | --- |
| TNF-α | GATCGGTCCCCAAAGGGATG | TGGTTTGTGAGTGTGAGGGT |
| NF-κB | GCCTCTGGCGAATGGCTTTA | TGCTTCGGCTGTTCGATGAT |
| GAPDH | TTCTCCTGCAGCCTCGT | ACGGCCAAATCTTGAGGTCT |

TNF-α: Tumor Necrosis Factor-alpha. NF-κB: Nuclear Factor kappa-light-chain-enhancer of activated B cells. GAPDH: Glyceraldehyde-3-phosphate dehydrogenase

Table S2. List of identified phenolic acids and flavonoids from the crude extract of Ficus altissima using HPLC by comparing with authentic standards.

| **No**. | **Compounds** | **M.F.** | **Rt. (min.)** | **Area %** |
| --- | --- | --- | --- | --- |
|  | **Polyphenolic derivatives** |  |  |  |
| 1 | Gallic acid | C_7_H_6_O_5_ | 3.566 | 8.34 |
| 2 | Chlorogenic acid | C_16_H_18_O_9_ | 4.231 | 28.87 |
| 3 | Methyl gallate | C₈H₈O₅ | 5.531 | 7.83 |
| 4 | Syringic acid | C₉H₁₀O₅ | 6.333 | 1.42 |
| 5 | Coumaric acid | C₉H₈O₃ | 8.585 | 2.52 |
| 6 | Vanillin | C₈H₈O₃ | 8.939 | 3.36 |
| 7 | Ferulic acid | C₁₀H₁₀O₄ | 9.531 | 1.0 |
| 8 | Rosmarinic acid | C₁₈H₁₆O₈ | 11.563 | 0.62 |
| 9 | Cinnamic acid | C₉H₈O₂ | 19.110 | 0.56 |
|  | **Flavonoids** |  |  |  |
| 10 | Rutin | C₂₇H₃₀O₁₆ | 6.911 | 4.71 |
| 11 | Naringenin | C₁₅H₁₂O₅ | 10.458 | 7.35 |
| 12 | Daidzein | C₁₅H₁₀O₄ | 15.640 | 0.45 |
| 13 | Quercetin | C₁₅H₁₀O₇ | 17.497 | 2.3 |
| 14 | Kaempferol | C₁₅H₁₀O₆ | 20.344 | 2.11 |

Rt. = retention time; M.F.= molecular formula; Area % = area percentage is refers to the relative proportion of each peak area compared to the total chromatographic area of all detected peaks.

Table S3. List of bioactive compounds identified from the crude extract of *Ficus altissima* using Gas Chromatography–Mass Spectrometry (GC–MS).

| **No**. | **Compounds** | **M.F** | **Rt. (min.)** | **Major Fragments**  **(m/z)** |
| --- | --- | --- | --- | --- |
|  | **Fatty acid derivatives** |  |  |  |
| 1 | Methyl-10,11-tetradecadienoate | C_15_H_26_O_2_ | 9.69 | 207, 177, 164, 150, 109, 95, 82, 74, 69, 67, 55 |
| 2 | Methyl 8,9-  Octadecadienoate | C_19_H_34_O_2_ | 10.06 | 294, 262, 196, 122, 109, 95, 81, 67, 55 |
| 3 | Methyl 12,13-tetradecadienoate | C_15_H_26_O_2_ | 13.49 | 206, 178, 123, 109, 95, 87, 81, 74, 67 |
| 4 | alpha linoleic acid, TMS derivative | C_21_H_38_O_2_Si | 25.07 | 335, 307, 291, 263, 149, 117, 95, 73, 55 |
| 5 | Stearic acid, TMS derivative | C_21_H_44_O_2_Si | 16.83 | 356, 341, 145, 132, 117, 73, 43 |
| 6 | Palmitic acid, TMS derivative | C_19_H_40_O_2_Si | 15.33 | 328, 313, 285, 269, 145, 129, 117. 73 |
| 7 | Myristic acid, TMS derivative | C_17_H_36_O_2_Si | 16.20 | 300, 285, 257, 185, 145, 129, 117, 37 |
|  | **Phenolics** |  |  |  |
| 8 | 4-hydroxy benzoic acid, 2TMS derivative | C_13_H_22_O_3_Si_2_ | 11.06 | 282, 267, 223, 193, 149, 126, 73 |
| 9 | vanillic acid, 2TMS derivative | C_14_H_24_O_4_Si_2_ | 12.58 | 312, 297, 282, 267, 253, 223, 193, 165, 126, 73 |
| 10 | protocatechuic acid, 3TMS derivative | C_16_H_30_O_4_Si_3_ | 13.19 | 355, 311, 281, 223, 193, 73 |
|  | **Sugar Derivatives** |  |  |  |
| 11 | Glucose, 5TMS derivatives | C_21_H_52_O_6_Si_5_ | 14.13 | 540, 507, 434, 419, 377, 361, 217, 191, 103, 147, 73 |
| 12 | Rhamnose, 4TMS derivative | C_18_H_44_O_5_Si_4_ | 11.36 | 393, 305, 217, 204, 191, 147, 73 |
| 13 | Xylose, 4TMS derivative | C_17_H_42_O_5_Si_4_ | 14.09 | 453, 365, 323, 217, 147, 37 |

M.F = Molecular formula. TMS derivatives = tetramethylsilane derivatives. Compound identification was achieved by analyzing retention times, mass spectra, and fragmentation pattern which were then compared to standard reference data from the Wiley and NIST libraries.


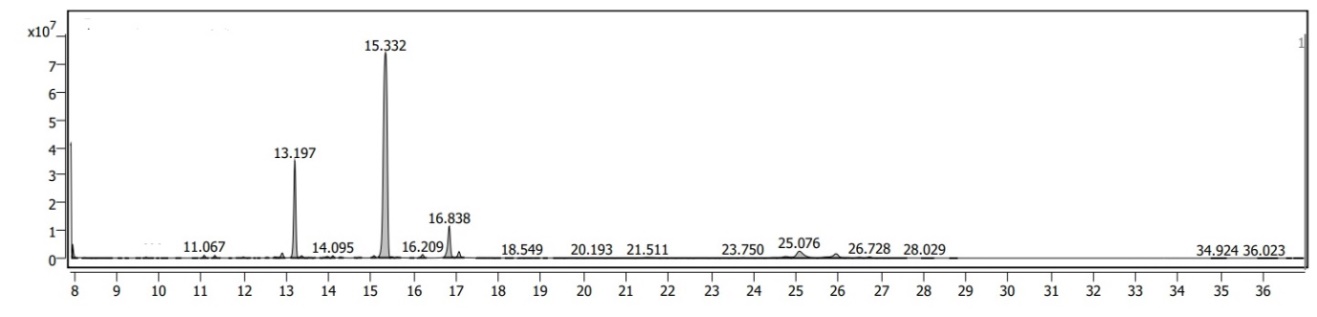


**Figure S1.** GC–MS total ion chromatogram (TIC) of the crude extract of *Ficus altissima*. Peaks are numbered according to their order of elution. Compound identification was performed by comparison of mass spectra, fragmentation pattern with those in the NIST library.

**Figure S2.** Calibration curve of gallic acid for the determination of total phenolic content (TPC) in the crude extract of *Ficus altissima*.

**Figure S3.** Calibration curve of quercetin (µg/mL) for the determination of total flavonoid content (TFC) in the crude extract of *Ficus altissima*.


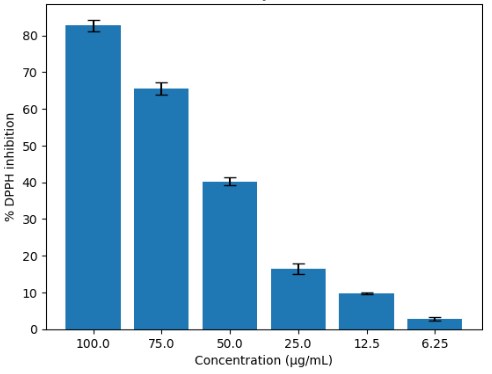


**Figure S4.** Antioxidant activity of Ficus altissima extract evaluated by the DPPH radical scavenging assay at different concentrations (6.25–100 µg/mL). Data are expressed as percentage of DPPH inhibition (mean ± SD, n = 3).


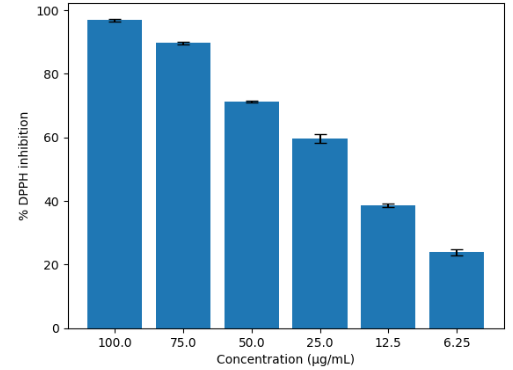


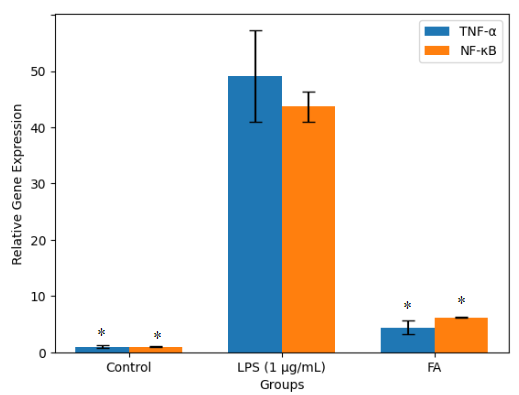
**Figure S5.** Antioxidant activity of ascorbic acid evaluated by the DPPH radical scavenging assay at different concentrations (6.25–100 µg/mL). Data are expressed as percentage of DPPH inhibition (mean ± SD, n = 3).

**Figure S6.** Effect of *F. altissima* crude extract on gene expression of TNF-α and NF-κB in LPS-treated RAW 264.7 cells. Control = untreated RAW 264.7 cells, LPS = Lipopolysaccharide-treated RAW 264.7 cells (1 µg/mL); FA = RAW 264.7 cells treated with F. altissima 100 µg/mL + LPS 1 µg/mL in RAW 264.7. data is presented as mean ±SD. Statistical significance was reported as p < 0.05, * indicates significance compared to the LPS group.
